# Fuzzy Logic: vulnerability of women who have sex with women to sexually transmitted infections

**DOI:** 10.1590/0034-7167-2023-0271

**Published:** 2024-07-29

**Authors:** Juliane Andrade, Kendra Yanne da Silva Santos, Ana Paula Freneda de Freitas, Mariana Alice Oliveira Ignácio, Emmanuel Zullo Godinho, Marli Teresinha Cassamassimo Duarte

**Affiliations:** I Universidade Estadual Paulista “Júlio de Mesquita Filho”, Faculdade de Medicina de Botucatu. Botucatu, São Paulo, Brazil; II Hospital Ortopédico e de Medicina Especializada. Brasília, Distrito Federal, Brazil; III Universidade Estadual Paulista “Júlio de Mesquita Filho”, Unidade Auxiliar da Faculdade de Medicina de Botucatu, Centro de Saúde Escola. Botucatu, São Paulo, Brazil; IV Centro Universitário Sagrado Coração, Departamento de Agronomia. Bauru, São Paulo, Brazil

**Keywords:** Fuzzy Logic, Sexually Transmitted Diseases, Women Who Have Sex With Women, Health Vulnerability, Reproductive Health, Lógica Difusa, Infecciones de Transmisión Sexual, Mujeres Que Tienen Sexo Con Mujeres, Vulnerabilidad de la Salud, Salud Reproductiva

## Abstract

**Objective:**

To describe the possibility of applying Fuzzy Logic in analyzing the vulnerability of Women Who Have Sex with Women to Sexually Transmitted Infections/HIV/AIDS.

**Methods:**

We developed a Fuzzy Logic system with 17 input variables and one output variable, using data related to vulnerability in a municipality located in the Midwest region of the State of São Paulo, Brazil.

**Results:**

The factor with the greatest positive impact was the confirmation that a low understanding of Sexually Transmitted Infections/HIV/AIDS is associated with higher vulnerability. Conversely, the statement “Not disclosing sexual activity to healthcare professionals,” where individuals do not admit to having sex with women, had the least impact.

**Conclusions:**

Fuzzy Logic facilitates the identification of vulnerability, expressed through the analysis of interaction between variables in each dimension. This makes it a promising method to assist in analyzing the vulnerability of specific populations.

## RESUMEN

**Objetivo:**

Describir la posibilidad de aplicación de la Lógica Difusa en el análisis de la vulnerabilidad de las Mujeres que tienen Relaciones Sexuales con Mujeres a las Infecciones de Transmisión Sexual/VIH/sida.

**Métodos:**

Se desarrolló un sistema de Lógica Difusa con 17 variables de entrada y una de salida, utilizando datos relacionados con la vulnerabilidad en un municipio ubicado en el Centro-Oeste del Estado de São Paulo, Brasil.

**Resultados:**

El factor de mayor impacto positivo fue la confirmación de que la baja comprensión sobre las Infecciones de Transmisión Sexual/VIH/sida está asociada con una mayor vulnerabilidad. Por otro lado, la declaración “No exponerse ante el profesional de la salud”, donde la persona no admite tener relaciones sexuales con mujeres, tuvo el menor impacto.

**Conclusiones:**

La Lógica Difusa permite la identificación de la vulnerabilidad, expresada por el análisis de interacción entre las variables de cada dimensión. Esto la convierte en un método prometedor para ayudar en el análisis de la vulnerabilidad de poblaciones específicas.

## INTRODUCTION

The Sexual and Reproductive Health (SRH) of women engaged in homosexual practices is still under-researched. It’s crucial to emphasize that sexual practices-homo, hetero, and bisexual-do not determine individuals’ sexual identity (lesbians, gays, and bisexuals). Therefore, to address the health needs of women practicing homo/bisexual behavior comprehensively, the term “women who have sex with women” (WSW) has emerged^(1)^.

In Brazil, lesbian women have been advocating for social and political visibility for decades through the Lesbian, Gay, Bisexual, Transvestite, and Transsexual (LGBT) movement, feminist lesbian activism, and their supporters. This advocacy has led to significant outcomes such as the National Policy for Comprehensive Women’s Health Care (PNAISM) ^(2)^, Basic Attention Booklets on Sexual Health and Reproductive Health^(3)^, the National Policy for Comprehensive LGBT Health^(4)^, among others. However, there are still numerous barriers and challenges to effectively implementing care actions in the routine of health services.

In the field of SRH, a focus on preventing Sexually Transmitted Infections/Human Immunodeficiency Virus/Acquired Immunodeficiency Syndrome (STIs/HIV/AIDS) is essential to establish care actions, alongside adopting comprehensive terminology to combat stigma and discrimination. From this perspective, there are several vulnerabilities for WSW, including lack of information on STI/HIV/AIDS prevention tailored to homosexual practices^(1,5)^, a heteronormative approach by health professionals often accompanied by discrimination and unpreparedness for care, gender disparities, breaches of patient confidentiality by professionals^(6)^, and inadequacy of STI/HIV/AIDS prevention measures recommended for female homosexual practices^(7)^.

Another factor contributing to the vulnerability of WSW to STIs/HIV/AIDS is the belief that they are immune to these infections^(5,7)^, held by both individuals and healthcare professionals, further underscoring the unpreparedness of professionals in this area. This belief is disproven when confronted with the prevalence of STIs/HIV/AIDS in WSW. A recent systematic review involving self-identified lesbian women or those reporting sexual practices with other women, regarding the risk of STIs/HIV/AIDS and bacterial vaginosis among the study population, indicated variable prevalence rates of curable or treatable STIs/HIV/AIDS in women residing in lowto middle-income countries, with rates of 2.4% for chlamydia, 0.6% for gonorrhea, 3.5% for trichomoniasis, and 0.5% for syphilis. The authors cited studies conducted in China, the United Kingdom, and Ireland, as well as another systematic review by Indian researchers, indicating HPV prevalence ranging from 11% to 15.6%^(8)^.

In this context, vulnerability is expressed through a combination of individual and collective aspects that heighten susceptibility to illness or health risks, directly tied to the availability of resources for health protection. Thus, vulnerability is categorized into three typically interconnected dimensions: individual, social, and programmatic^(9)^.

The individual dimension encompasses personal resources such as access to information on STIs/HIV/AIDS and personal support networks, as well as intersubjective subjectivity, including values, beliefs, and desires, which may or may not conflict. The social dimension involves gender relations, intergenerational, economic, racial, and ethnic relationships, processes of stigmatization, poverty, and social exclusion. The programmatic dimension assesses the extent to which government institutions protect and promote the right to health^(9)^.

To analyze the vulnerability of MSM, the use of Fuzzy Logic can serve as an alternative, as it has been applied in the health field and can manage linguistic information from the variables under study, taking into account expert opinions on the observed phenomenon, as well as handling large datasets in an accessible manner, thus facilitating decision-making^(10)^.

There are several theories surrounding Fuzzy Logic, with the primary one involving the management of uncertainties and ambiguities as deterministic numerical values^(11)^. Recognizing that the degree of uncertainty is referred to as the membership value, Fuzzy Logic operates with data and results closer to non-mathematical language and more toward humanistic language, as illustrated by examples such as “it may rain” or “that man is tall”^(12)^.

Given the scarcity of studies on the presented topic and the urgency of research providing support for the effectiveness of health policies in delivering quality care for this population, as well as the utilization of Fuzzy Logic-based systems to aid decision-making, the following study was proposed. This study complements the article titled “Vulnerability of women who have sex with women to sexually transmitted infections”^(13)^, aiming to develop a model based on fuzzy rules that assess the effects of variations in social, individual, and programmatic vulnerability in the analysis of MSM vulnerability to STIs/HIV/AIDS.

## OBJECTIVE

To describe the potential application of Fuzzy Logic in analyzing the vulnerability of Women Who Have Sex with Women to Sexually Transmitted Infections/HIV/AIDS.

## METHODS

### Ethical aspects

The main study that supported this research was submitted and approved by the Research Ethics Committee of FMB-UNESP and received a favorable opinion (no. 820,717) on 20/10/2014. All participants were adequately informed about the objectives and manner of participation, and for those who agreed, they were asked to sign the Informed Consent Form.

### Study design

This study outlines the methodological approach to data analysis using Fuzzy Logic applied to a comprehensive research dataset^(14)^, aimed at assessing access to healthcare services and SRH among MSM, described based on the Strengthening the Reporting of Observational Studies in Epidemiology (STROBE) tool.

### Place

It was conducted in the municipality of Botucatu, located in the Midwest region of the State of São Paulo.

### Methodological procedures

To simulate human thinking, variables indicating greater vulnerability to STIs/HIV/AIDS in healthcare practice and according to scientific knowledge were designated by three specialists in the field and in accordance with scientific literature^(15-17)^. The specialists were recruited for their extensive experience in healthcare and research on STIs/HIV/AIDS among MSM.

The input variables of the proposed Fuzzy classification system were divided into three groups, each group having a certain number of input variables, according to the vulnerability framework (individual, social, and programmatic) ^(9)^ and listed considering data availability.

In the individual vulnerability group, the following variables were included: age ≤ 24 years; two or more sexual partnerships in the last 12 months; lack of basic knowledge about STIs/HIV/AIDS; engaging in sex in exchange for money and/or drugs; absence of serological tests for STIs/HIV/AIDS; sexual relations with men in life; lack of risk perception for STIs/HIV/AIDS; sexual relations with men in the last 12 months; tribadism (sexual practices between women); sex after consuming illicit drugs and/or alcohol; and sexual intercourse during menstruation^(13)^.

Variables in the social vulnerability group include: per capita income < R$291.00 and not declaring having sex with women at healthcare services^(13)^.

**Figure 1:**
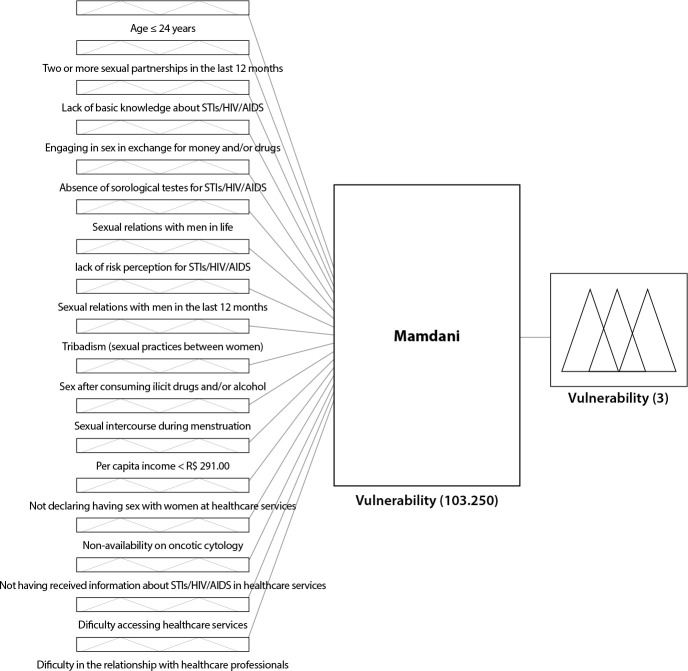
Structure of the proposed Fuzzy system

Finally, the variables of programmatic vulnerability include: the non-availability of oncotic cytology; not having received information about STIs/HIV/AIDS in healthcare services; difficulty accessing healthcare services, and difficulty in the relationship with healthcare professionals^(13)^. Figure 1 depicts the structure of the proposed Fuzzy system, comprising the input interface, the rules block, and the output interface. The connecting line symbolizes the flow of data. Thus, all possibilities of the input variables activate rules in the rules block, resulting in the possibilities described in the final output variable.

The system was developed based on a correlation of “no” and “yes” responses for each membership data (individual, social, and programmatic), with each group having a certain number of variables, as described earlier, with 11 in the individual group, 2 in the social group, and 4 in the programmatic group.

**Figure 2:**
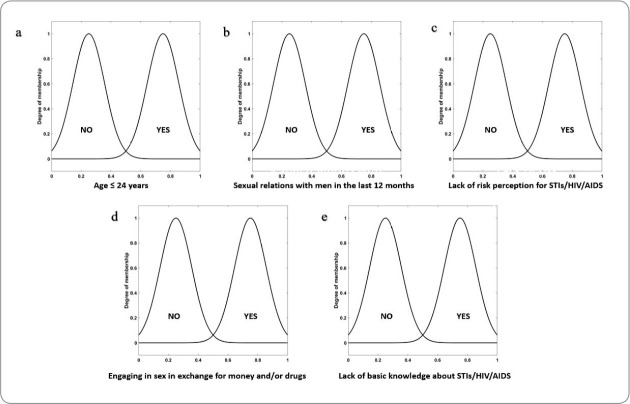
Examples of five of the 17 input variables of the developed model

Thus, responses that are below or equal to 50% would have a “no” membership data, and those above 50% would have a “yes” membership data. The graphs were developed in Gaussian format due to their undulations, facilitating the visualization of variable changes within the structure, i.e., from maximum to minimum points, in addition to the critical importance of the structure, as studied using this method. Figure 2 shows five examples of input data from the 17 graphs that were constructed.

**Figure 3:**
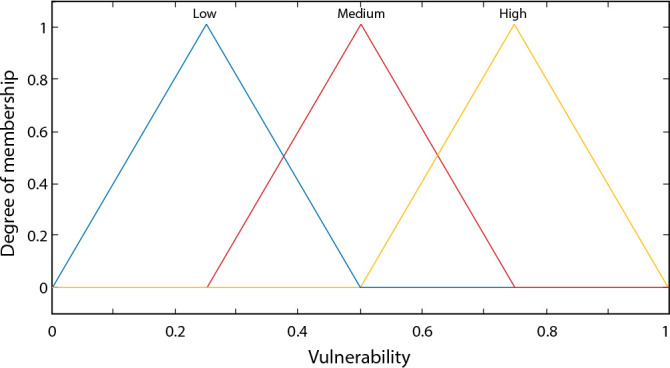
Membership function of the output variable “Vulnerability”

The output variable was named “Vulnerability” and is presented in the proposed system with the following membership data: Low [0.0-0.5], Medium [0.25-0.75], and High [0.5-1.0], as depicted in Figure 3. This result is similar to what a nursing professional could obtain with their field experience, but here it is supported by Fuzzy Logic.

In this context, Fuzzy Logic, combined with the theoretical framework of vulnerability by Ayres et al. ^(9)^, enables a deeper understanding of MSM vulnerability to STIs/HIV/AIDS, with the aim of optimizing various variables applied in the patient’s daily life. Therefore, the application of Fuzzy Logic was proposed in the form of a Case Study, which included a participant from the main study.

### Data Collection and Organization

Data from the main study were collected through the administration of a questionnaire specifically designed for the research, gynecological examination, and peripheral blood collection, as detailed in Andrade et al. ^(13)^. The target population of the study comprised women who reported having sex with women or both women and men, over 18 years old, residing in municipalities belonging to the Regional Department of Health of Bauru (DRS-VI). Extensive dissemination was conducted for participant recruitment. The offered exams aimed to detect the diagnosis of any STI: Chlamydia trachomatis, Neisseria gonorrhoeae, Trichomonas vaginalis, Human Papillomavirus (HPV) infections, Human Immunodeficiency Virus (HIV), hepatitis B, or syphilis.

### Analysis of data

The Mamdani inference method^1 2^ was utilized to calculate the numerical values of the input variables in relation to the output variable using Matlab 2022b software, in order to construct a system based on computational Fuzzy rules and develop plots and contour maps of the system’s representation function. It is noteworthy that the software used is licensed by the AGROENERBIO research group - Energy and simulation in biosystems engineering and agribusiness, located at USP/FZEA in Pirassununga SP.

The Mamdani method yields a Fuzzy set as a response, derived from the combination of input values with their respective degrees of membership, through the minimum operator and then by the overlap of rules by the maximum operator^(12)^. For the defuzzification process, the Center of Area (C-o-A) was employed, returning the center of the area under the developed curve, as utilized by other authors^(11)^.

## RESULTS

The total number of rules was determined by the number of possible combinations for the responses of the 17 variables for each woman, considering that each variable has the option of two alternatives: “no” or “yes”. Thus, the rule bases establish relationships between the input variables and the output variable through propositions of the type “IF” and “THEN”. In this manner, a total of 103,250 correlations were computed, with these combinations yielding the system’s response (Table 1).

**Table 1:** Rule base of the Fuzzy system

	1	2	3	4	...	8	9	10	11	12	13	14	15	16	17	1
**Id**	**VE**	**VE**	**VE**	**VE**	...	**VE**	**VE**	**VE**	**VE**	**VE**	**VE**	**VE**	**VE**	**VE**	**VE**	**VS**
1	Yes	Yes	Yes	Yes	Yes	Yes	Yes	Yes	Yes	Yes	Yes	Yes	Yes	Yes	Yes	B
2	Yes	Yes	Yes	Yes	Yes	Yes	Yes	Yes	Yes	Yes	Yes	Yes	Yes	Yes	No	M
3	Yes	Yes	Yes	Yes	Yes	Yes	Yes	Yes	Yes	Yes	Yes	Yes	Yes	Yes	Yes	A
4	Yes	Yes	Yes	Yes	Yes	Yes	Yes	Yes	Yes	Yes	Yes	Yes	Yes	Yes	No	B
5	Yes	Yes	Yes	Yes	Yes	Yes	Yes	Yes	Yes	Yes	Yes	Yes	No	No	Yes	B
6	Yes	Yes	Yes	Yes	Yes	Yes	Yes	Yes	Yes	Yes	Yes	Yes	No	No	No	M
7	Yes	Yes	Yes	Yes	Yes	Yes	Yes	Yes	Yes	Yes	Yes	Yes	No	No	Yes	M
8	Yes	Yes	Yes	Yes	Yes	Yes	Yes	Yes	Yes	Yes	Yes	Yes	No	No	No	B
...	...	...	...	...	...	...	...	...	...	...	...	...	...	...	...	...
103.249	No	No	No	No	No	No	No	No	No	No	No	No	No	No	No	M
103.250	No	No	No	No	No	No	No	No	No	No	No	No	No	No	Yes	M

*Notes: id: Identification; IV - Input variable; OV - Output variable*

The chosen approach involved formulating rules that encompassed all possible combinations of input variables with respect to the output variable. For instance, in the initial row of Table 1, the input variables were interconnected and yielded “yes” responses, leading to a “Low” output variable, and so forth.

**Figure 4:**
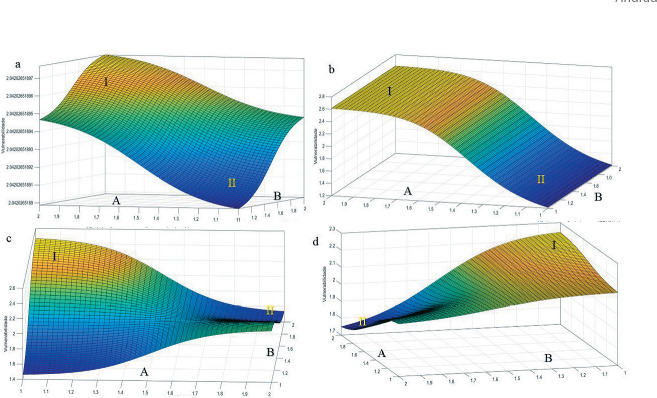
3D surface models depicting Vulnerability responses to the 17 input variables

Using the developed model, it became feasible to generate 3D surface responses for vulnerability and corresponding contour maps. These visualizations confirmed the actual inference of the 17 input variable blocks. The rule-based Fuzzy model systematically assessed all potential combinations of input variables across two levels (“no” and “yes”), resulting in a rule base comprising 103,250 combinations, which corresponds to the factorial of 2^17. This is depicted in the response surface models illustrated in Figure 4.

*Caption: (Figure a): A: Not declaring having sex with women at healthcare services x B: Per capita income < R$291.00; (Figure b): A: Sexual relations with men in the last 12 months x B: Lack of risk perception for STIs/HIV/AIDS; (Figure c): A: Lack of basic knowledge about STIs/HIV/AIDS x B: Engaging in sex in exchange for money and/or drugs; (Figure d): A: Two or more sexual partnerships in the last 12 months x B: Per capita income < R$291.00.*

It was understood (Figure 4) that the indicator that most responded to high vulnerability was when the person had low knowledge. In Figure 4, this indicator represented an intermediate value between 0.5 and 1.0, with 1.0 being the maximum point. The least impactful indicator on a person’s vulnerability in this modeling was the declaration of not exposing oneself to healthcare professionals with 0.1498, best exemplified when the person does not disclose having sex with women to the healthcare professional.

In more detail, Figure (4a) presented two important regions, with I being the point with the highest vulnerability when evaluating the input variables (not disclosing having sex with women at healthcare services x per capita income < R$291.00), as both had responses at the highest point (2.0), resulting in both variables being determinants for responding to high vulnerability. In indicator II, the opposite occurred, with lower points indicating lower chances of vulnerability. It is important to note that in this Figure (a), both variables have a preponderance for high vulnerability when the characterization points are close to 100%.

Figure (4b) presented the relationship between the variables sexual relations with men in the last 12 months x lack of risk perception for IST/HIV/AIDS, with point I indicating higher vulnerability due to not having the risk perception for IST/HIV/AIDS, as it presented both “no” and “yes” at strong points, while for sexual relations with men in the last 12 months, the values were above 80% for “yes”. A striking characteristic in this figure was that not having risk perception for IST/HIV/AIDS was considered the factor that most impacts vulnerability. For point II, the same occurred for the variable of not having risk perception for IST/HIV/AIDS and a lower indicator for the variable of sexual relations with men in the last 12 months.

For Figure (4c), high vulnerability marked at point I, with a high indicator of “yes” for engaging in sex in exchange for money and/or drugs, with a lower indicator of lack of basic knowledge about STIs/HIV/AIDS. The lowest vulnerability indicator was having extensive knowledge about the two related variables. This Figure (4c) did not have the characteristics of the previous figures (a and b), as both did not represent a high relationship to vulnerability.

In Figure (4d), the indicators of high and low vulnerability (I and II) were presented, respectively, with point I having the highest indicator represented for per capita income < R$291.00, as it can lead to a lack of important information for illness prevention. When evaluating point II, both indicators were at the highest points, resulting in low vulnerability, when the variables of two or more sexual partnerships in the last 12 months x per capita income < R$291.00 were applied.

## DISCUSSION

It was possible to apply Fuzzy Logic, following the step-by-step process exemplified in a case study, to determine and support, with scientific data, a potential vulnerability of MSM to STIs/HIV/AIDS. The use of artificial intelligence-based tools, as proposed in this article, brings contributions to all involved in this issue, whether they are healthcare professionals or users of the healthcare system. As this is a pilot study, it serves as an incentive for the development of software that facilitates the analysis of data related to the topic.

Fuzzy Logic as a basis for creating healthcare technology enables decision-making and problem-solving in complex individual and collective care contexts. It adds to this comprehensiveness through the articulation with other healthcare sectors, and effectiveness through planning and achieving goals according to the analysis of health needs of the patient, the specific population to which she belongs, or the general population, thus contributing to minimizing aspects of programmatic vulnerability^(18)^.

Statistical analyses transform numbers into information. In this article, we chose to use Fuzzy Logic to directly address the information inherent to human language, transforming patients’ responses into situations that portray their vulnerability. Although this study deals with binary responses, “no” or “yes,” it is possible to incorporate different responses into the Fuzzy Logic software to make the analysis more accurate, since, to express human language, one must understand the uncertain nuances that exist between “no” and “yes.” This is a research that illustrates the use of Fuzzy Logic for vulnerability analysis. Therefore, the importance of this article for the MSM population is highlighted, considering the challenges they still face, especially regarding their invisibility in healthcare services and the lack of professional preparedness in caring for this population^(13)^.

Thus, the proposed information system can be transformed into a mobile application and/or computer software, so that it can be used even in a prognostic and diagnostic manner. Healthcare technologies supported by Fuzzy Logic have already been used in various areas of healthcare, such as monitoring children and adolescents with chronic diseases through a Decision Support Expert System^(19)^, and in determining the degree of health risk for pregnant women^(20)^.

It is recommended, furthermore, before the development of the application, to conduct a cross-sectional study applying Fuzzy Logic to the group of women included in the larger study^(14)^ in order to operationalize the application of the method presented here.

### Limitations of the Study

A limitation of the study is highlighted by the fact that the variables have binary responses (“yes” or “no”), which do not fit within the nature of the research, as responses such as “maybe,” “much,” or “little” are not included. This is a condition that could be explored in Fuzzy Logic. Additionally, limitations of the study include the method of selecting input variables for convenience, noting that numerous other vulnerability conditions may coexist, increasing the vulnerability of MSM to STIs/HIV/AIDS.

### Contributions to Nursing and Public Health

The possibility of practical application of the vulnerability concept, through the use of Fuzzy Logic supported by information technologies, contributes to the reflection on nursing care in public health.

## CONCLUSIONS

Fuzzy Logic can provide an opportunity for analyzing vulnerability expressed by the interaction between the variables of each dimension, proving to be a viable analysis method for specific populations. It is suggested to develop studies with larger population samples that can improve the model and guide the development of technologies that facilitate decision-making in real scenarios of professional practice.

## Supplementary Material

0034-7167-reben-77-03-e20230271-suppl01

## Data Availability

https://hdl.handle.net/11449/255136
