# Supplementary material for: Fuzzy Logic: vulnerability of women who have sex with women to sexually transmitted infections
Source: Rev Bras Enferm. 2024 Jul 29;77(3):e20230271. doi: 10.1590/0034-7167-2023-0271 (PMC11290739; doi:10.1590/0034-7167-2023-0271)
Supplement: 0034-7167-reben-77-03-e20230271-suppl01 [file 0034-7167-reben-77-03-e20230271-suppl01.pdf]

CODNOVO	IND_ANS	ID	BRANCA	ESC	UES	AR	RPC	REL	VI1	VI2	VI3	VI4	VI5	VI6	VI7	VI8	**V9N**	**V10N**	VI11	VI12	VI13
2	1	26	0	0	1	0	466.67	0	0	1	0	0	0	1	0	0	1	1	0	1	0
3	1	20	1	0	0	1	1086.00	1	1	0	0	1	0	0	0	1	1	1	1	1	1
5	1	37	1	1	0	1	500.00	1	0	0	0	1	0	1	0	0	1	1	1	1	0
6	1	34	1	1	0	1	3000.00	1	0	0	0	1	0	0	0	0	1	1	0	0	0
7	1	28	1	0	1	1	1050.00	1	0	0	0	0	1	0	0	0	1	1	0	0	0
8	1	30	1	0	1	1	1050.00	1	0	0	0	0	0	0	0	0	1	1	0	0	0
9	1	22	1	1	0	1	750.00	1	1	0	0	1	0	0	0	1	1	1	1	1	1
10	1	42	1	0	0	0	750.00	1	0	0	0	1	0	0	0	0	0	0	1	0	0
11	1	29	1	1	1	1	1000.00	1	0	0	0	0	0	0	0	0	0	0	1	0	0
12	1	34	1	1	1	1	1750.00	1	0	0	0	0	0	0	1	0	1	1	1	1	1
13	1	18	0	0	0	0	133.33	0	1	1	0	1	0	0	0	1	0	0	1	0	0
15	1	33	0	0	1	1	1250.00	1	0	1	0	0	0	0	0	0	1	1	1	0	0
16	1	21	1	0	1	1	325.00	0	1	0	0	0	0	0	0	1	0	0	1	0	0
18	1	22	1	0	1	1	375.00	1	1	0	0	0	1	0	0	1	1	1	1	0	0
19	1	28	0	0	0	1	420.00	0	0	1	0	1	1	1	0	0	0	1	0	0	0
21	1	28	1	1	1	1	1750.00	1	0	0	0	0	0	0	0	0	1	1	0	1	0
22	1	19	1	1	0	1	2666.67	0	1	0	0	1	0	0	0	1	0	1	1	0	1
23	1	32	1	1	0	1	1750.00	0	0	0	0	1	0	0	0	0	1	1	0	0	0
24	1	41	1	1	0	1	3500.00	1	0	0	0	1	0	0	0	0	1	1	0	0	0
25	1	32	1	1	0	0	666.67	1	0	0	0	1	0	0	0	0	0	0	0	0	0
26	1	37	1	0	0	1	1900.00	1	0	0	0	1	0	0	0	1	0	0	1	0	1
27	1	34	0	0	0	1	900.00	1	0	1	0	1	0	1	0	1	0	0	1	0	0
28	1	30	0	0	0	1	276.00	1	0	1	0	1	0	1	1	0	1	1	0	1	0
29	1	19	1	1	0	1	1500.00	1	1	0	0	1	1	0	1	1	1	1	0	0	0
30	1	25	1	1	0	0	4000.00	0	0	0	0	1	1	0	1	0	0	1	1	0	1
31	1	30	1	1	1	1	1333.33	1	0	0	0	0	0	0	1	0	0	0	0	0	0
32	1	24	0	1	0	1	1576.00	1	1	1	0	1	1	0	0	1	1	0	1	0	0
33	1	52	1	0	0	1	700.00	1	0	0	0	1	1	0	0	1	0	0	0	0	0
34	1	60	0	1	1	0	1000.00	1	0	1	0	0	0	0	0	1	1	1	0	0	0
35	1	24	1	1	0	1	2000.00	0	1	0	0	1	0	0	0	0	1	1	1	0	1
36	1	21	1	0	1	1	583.33	1	1	0	0	0	0	1	1	0	1	1	1	0	0
37	1	22	1	1	0	1	1000.00	0	1	0	0	1	0	0	0	0	0	1	1	0	0
38	1	52	1	0	1	1	1200.00	1	0	0	1	0	0	0	0	0	1	1	1	0	0
39	1	47	1	1	1	1	1250.00	1	0	0	0	0	0	0	0	1	1	1	0	0	0
40	1	29	1	0	0	1	181.20	1	0	0	0	1	1	1	0	0	1	1	1	0	0
41	1	18	0	0	0	0	1047.00	0	1	1	0	1	0	1	0	1	1	1	1	1	0
42	1	21	1	1	0	0	666.67	1	1	0	0	1	0	0	0	0	1	1	1	0	0
43	1	20	1	1	0	0	1050.67	1	1	0	0	1	0	0	0	0	1	1	1	0	0
44	1	30	1	1	1	1	1050.00	1	0	0	0	0	0	0	0	0	1	1	0	0	0
45	1	28	1	0	1	1	666.67	1	0	0	0	0	0	0	0	0	0	0	1	0	0
46	1	20	1	1	0	1	788.00	1	1	0	0	1	0	0	0	0	1	1	0	0	0
47	1	40	0	0	0	1	1000.00	0	0	1	0	1	0	1	0	0	1	1	1	0	0
48	1	37	1	0	1	1	433.33	1	0	0	0	0	0	0	0	1	1	1	1	0	0
49	1	18	1	0	0	1	1454.67	0	1	0	0	1	1	0	0	0	0	0	1	0	0
50	1	49	1	0	1	1	2100.00	1	0	0	1	0	0	1	0	0	0	1	1	0	0
51	1	42	0	0	0	1	1500.00	1	0	1	0	1	0	1	0	0	0	1	1	1	0
52	1	27	1	1	0	1	6666.67	1	0	0	0	1	0	1	0	0	0	1	1	1	0
53	1	24	0	0	0	1	900.00	1	1	1	0	1	1	0	0	1	1	1	1	0	0
54	1	19	1	0	0	1	600.00	0	1	0	0	1	0	0	0	1	1	1	1	1	0
55	1	20	1	0	0	0	525.00	0	1	0	0	1	0	0	0	1	0	1	1	1	0
56	1	18	1	0	0	0	472.80	1	1	0	0	1	0	0	1	1	0	1	0	1	0
57	1	34	1	0	0	1	1500.00	1	0	0	0	1	1	0	1	1	1	1	1	1	1
58	1	19	1	0	0	0	1333.33	0	1	0	0	1	1	0	0	1	1	1	1	1	1
59	1	26	0	0	1	0	1600.00	1	0	1	0	1	0	0	0	0	0	1	0	0	0
61	1	50	1	0	1	0	500.00	1	0	0	1	0	0	0	0	0	0	1	0	1	0
63	1	52	1	1	0	1	1666.67	1	0	0	0	1	0	0	0	0	1	1	0	0	0
64	1	22	1	0	0	1	3000.00	1	1	0	1	1	1	0	0	1	1	1	1	0	0
65	1	26	1	0	1	1	452.50	1	0	0	1	0	1	0	0	0	0	0	0	1	0
66	1	23	0	0	1	1	350.00	0	1	1	0	0	1	0	0	0	0	1	0	0	0
68	1	31	0	1	0	1	1600.00	0	0	1	0	1	0	0	0	0	1	1	1	1	1
69	1	18	1	0	0	0	1125.00	1	1	0	0	1	1	1	0	0	0	0	1	0	0
70	1	21	1	1	0	1	788.00	0	1	0	0	1	0	0	0	0	0	0	1	0	0
72	1	33	1	1	0	1	3000.00	0	0	0	0	1	0	0	0	1	0	0	1	0	1
73	1	18	1	0	1	0	533.33	1	1	0	0	0	0	0	1	1	0	1	1	1	1
74	1	52	0	0	1	1	1838.67	1	0	1	0	0	0	1	0	0	1	1	1	0	0
75	1	49	1	1	1	1	4000.00	1	0	0	0	0	0	0	0	1	1	1	0	0	0

76	1	20	1	1	0	0	2000.00	0	1	0	0	1	0	1	0	0	1	1	0	0	0
77	1	43	1	0	1	0	800.00	1	0	0	0	0	0	0	1	0	0	0	0	0	0
80	1	30	1	0	0	1	575.00	1	0	0	0	1	1	1	0	1	0	0	1	0	0
81	1	21	1	0	0	1	1000.00	1	1	0	0	1	0	0	0	1	1	1	1	1	1
82	1	27	0	0	1	1	475.00	1	0	1	0	0	0	0	1	0	0	0	1	0	0
84	1	20	0	0	0	1	1044.00	1	1	1	0	1	0	0	0	1	1	1	1	1	0
85	1	36	1	0	0	1	2000.00	1	0	0	0	1	1	0	0	1	1	1	1	0	0
86	1	25	1	0	1	1	1050.00	1	0	0	0	0	0	0	0	0	1	1	1	1	1
87	1	28	1	1	1	1	1500.00	1	0	0	0	0	0	0	0	1	1	1	1	1	1
88	1	20	1	0	1	0	500.00	0	1	0	0	0	1	1	0	1	1	1	0	0	1
89	1	31	1	1	0	1	2200.00	1	0	0	0	1	0	1	0	1	0	0	1	0	0
90	1	24	0	0	1	1	1300.00	1	1	1	0	0	0	0	0	1	1	1	1	0	0
92	1	25	0	1	0	1	1500.00	1	0	1	0	1	1	1	0	1	1	1	1	0	0
93	1	29	0	0	0	1	2000.00	1	0	1	0	1	1	1	0	1	1	1	1	0	0
94	1	20	1	1	0	1	1000.00	1	1	0	0	1	1	0	0	1	0	1	0	1	1
95	1	33	0	0	0	1	1000.00	1	0	1	0	1	0	0	0	1	1	1	0	0	0
96	1	36	1	1	0	1	1500.00	0	0	0	0	1	0	1	1	1	1	1	0	0	0
97	1	21	1	0	0	1	533.33	0	1	0	0	1	1	0	0	1	0	0	0	0	1
98	1	25	1	1	0	1	1000.00	1	0	0	0	1	1	1	0	0	1	1	1	0	1
99	1	33	1	1	1	1	1200.00	0	0	0	0	0	0	0	0	0	1	1	1	0	0
100	1	38	1	1	1	1	900.00	0	0	0	0	0	0	0	0	0	1	1	1	0	0
101	1	46	1	1	1	1	1666.67	1	0	0	0	0	1	0	0	1	1	1	0	1	0
102	1	46	1	1	1	1	1000.00	1	0	0	0	0	1	0	0	0	1	1	1	1	0
104	1	32	1	1	0	1	1000.00	1	0	0	0	1	0	0	0	0	1	1	0	0	0
105	1	32	1	1	0	1	237.50	1	0	0	0	1	1	0	1	0	1	1	1	1	1
106	1	20	1	1	0	0	375.00	1	1	0	0	1	0	0	0	0	0	0	1	0	1
107	1	23	1	1	0	0	600.00	1	1	0	0	1	0	0	0	0	1	1	1	0	1
108	1	20	0	0	0	1	340.00	1	1	1	0	1	1	0	0	1	0	0	1	0	1
109	1	21	0	1	0	1	1000.00	1	1	1	0	1	1	0	0	1	1	1	1	1	1
110	1	29	1	0	0	1	1666.67	1	0	0	0	1	0	0	0	0	0	0	1	0	0
111	1	24	1	1	0	1	1750.00	1	1	0	0	1	1	0	0	1	0	0	1	0	0
112	1	20	0	1	0	0	375.00	1	1	1	0	1	1	0	0	1	0	0	0	0	0
113	1	23	0	1	0	0	500.00	1	1	1	0	1	1	0	0	1	1	1	1	0	1
114	1	28	1	1	0	1	3500.00	1	0	0	0	1	1	0	0	0	1	1	1	0	0
115	1	19	1	0	0	1	320.00	0	1	0	0	1	0	0	0	0	0	0	1	0	0
116	1	19	1	1	0	0	750.00	0	1	0	0	1	0	0	0	0	0	0	1	1	1
117	1	21	1	0	1	0	500.00	1	1	1	0	1	1	0	0	1	1	1	1	0	0
118	1	19	1	1	0	1	750.00	0	1	0	0	1	1	0	0	0	0	0	1	1	0
119	1	37	0	0	0	1	666.67	1	0	1	0	1	1	0	0	1	0	0	1	0	0
120	1	23	0	0	0	1	950.00	1	1	1	0	1	1	0	0	1	1	1	1	0	0
121	1	27	1	1	0	1	1666.67	1	0	0	0	1	1	0	0	1	1	1	1	0	0
122	1	32	1	1	0	1	2500.00	1	0	0	0	1	1	0	0	0	1	1	1	0	0
123	1	27	1	1	0	1	1500.00	1	0	0	0	1	1	0	0	0	1	1	1	1	1
124	1	20	1	1	0	0	5000.00	1	1	0	0	1	0	0	1	0	1	1	1	0	0
125	1	20	0	1	0	0	2000.00	0	1	1	0	1	0	0	0	0	0	1	1	0	1
126	1	20	1	1	0	0	1500.00	1	1	0	0	1	1	0	1	0	0	0	1	0	1
127	1	18	1	1	0	1	750.00	0	1	0	0	1	1	0	0	1	0	0	1	0	1
128	1	22	1	0	0	0	3500.00	1	1	0	0	1	0	0	0	0	1	1	0	1	0
129	1	23	1	0	0	1	1300.00	1	1	0	0	1	1	0	0	0	1	1	1	0	0
130	1	30	1	0	0	1	1000.00	1	0	0	0	1	1	0	0	1	0	0	1	0	0
131	1	21	1	1	0	1	600.00	0	1	0	0	1	1	0	0	1	0	1	1	0	1
132	1	22	1	1	0	0	1800.00	0	1	0	0	1	0	0	0	0	0	0	1	0	1
133	1	33	1	0	0	1	1500.00	1	0	0	0	1	1	1	0	0	1	1	1	0	0
134	1	21	1	0	0	1	1000.00	0	1	0	0	1	0	0	0	1	0	0	1	0	0
135	1	24	0	0	0	1	900.00	1	1	1	0	1	0	0	0	1	0	0	1	0	0
136	1	31	1	1	1	1	1800.00	1	0	0	0	0	1	0	0	1	1	1	1	0	0
137	1	18	0	1	0	0	500.00	1	1	1	0	1	1	0	0	1	1	1	0	0	0
138	1	25	0	1	0	0	600.00	1	0	1	0	1	0	0	0	1	0	0	1	1	1
139	1	21	1	0	0	0	1500.00	1	1	0	0	1	1	0	0	0	0	0	1	1	1
140	1	25	0	0	0	0	800.00	1	0	1	0	1	1	0	0	1	0	0	1	0	0
141	1	24	1	1	0	1	2000.00	1	1	0	0	1	0	0	0	1	0	0	1	0	1
142	1	41	0	0	0	1	1000.00	1	0	1	0	1	1	0	0	0	1	1	1	0	0
144	1	20	1	1	0	0	1750.00	1	1	0	0	1	1	0	0	0	0	1	0	0	0
145	1	19	1	0	0	0	600.00	1	1	0	0	1	1	0	0	1	0	0	1	1	1
146	1	29	1	1	1	1	1750.00	0	0	0	0	0	1	0	0	1	0	0	1	0	0
147	1	20	1	1	0	0	5000.00	0	1	0	0	1	0	0	0	1	0	0	1	0	1
148	1	27	1	1	1	1	1250.00	1	0	0	0	0	1	0	0	0	1	1	1	0	0

149	1	37	0	1	1	1	600.00	1	0	1	0	0	1	0	0	1	1	1	0	0	0
150	1	25	1	0	0	1	3500.00	1	0	0	0	1	0	0	0	1	1	1	1	0	0
151	1	23	1	1	0	0	400.00	1	1	0	0	1	0	0	0	0	1	1	0	0	0
153	1	30	1	1	0	0	2250.00	1	0	0	0	1	0	0	0	0	0	0	1	0	1
154	1	45	1	1	0	1	1750.00	0	0	0	0	1	0	1	0	0	1	1	1	0	0
156	1	42	1	0	1	1	486.70	1	0	0	1	0	1	1	0	0	1	1	1	1	0
157	1	26	1	0	1	1	1600.00	1	0	0	0	0	0	1	0	0	1	1	0	0	0
159	1	25	1	0	0	1	640.00	1	0	0	0	1	1	0	0	1	0	0	1	0	1
160	1	23	0	0	0	1	500.00	1	1	1	0	1	1	0	0	1	1	1	0	0	1
161	1	27	0	1	0	0	225.00	0	0	0	0	1	1	0	0	0	0	0	1	0	1
162	1	62	1	1	0	1	15000.00	1	0	0	0	1	1	0	0	1	0	0	0	0	0
163	1	55	1	1	0	1	2500.00	1	0	0	0	1	1	0	0	1	1	1	0	0	1
164	1	62	1	1	0	1	10000.00	1	0	0	0	1	0	0	1	0	0	0	1	0	0
165	1	29	1	1	0	1	666.66	1	0	0	0	1	0	1	0	0	1	1	1	0	0
168	1	22	1	1	0	0	833.33	1	1	0	0	1	1	1	0	1	1	1	1	0	1
166	1	30	0	1	1	1	3000	1	0	1	0	1	1	1	0	1	0	0	1	0	0
167	1	27	1	1	0	1	1200	0	0	0	0	1	0	0	0	0	1	1	1	0	0

VI14	VI15	VI16	VI17	VI18	VI19	VI20	VI21	VI23	VI24	VI25	ESCVI	ESCVIp	VS1	VS2	VS3	VS4	VS5	ESCVS	ESCVSp	ESCSp2	VP1
0	1	0	1	0	0	0	0	1	1	0	9	-0.17	1	0	0	1	0	2	0.51	9.60	0
1	1	0	0	0	0	0	0	1	0	0	11	0.52	0	0	1	1	0	2	0.51	9.60	0
1	0	1	1	1	1	0	0	1	1	1	14	1.57	0	0	1	1	0	2	0.51	9.60	0
0	0	0	1	0	1	0	0	0	1	0	6	-1.21	0	0	1	0	0	1	-0.53	4.80	0
0	1	0	1	0	1	0	0	0	0	0	6	-1.21	0	0	0	1	1	2	0.51	9.60	0
0	0	0	1	0	1	0	0	0	1	0	5	-1.56	0	0	1	1	0	2	0.51	9.60	0
1	0	0	1	1	1	0	1	1	1	0	15	1.91	0	0	0	1	0	1	-0.53	4.80	0
0	0	0	1	0	1	0	0	1	1	0	6	-1.21	1	0	0	0	0	1	-0.53	4.80	0
0	1	0	1	0	1	0	1	0	0	0	5	-1.56	0	0	0	1	0	1	-0.53	4.80	1
1	0	0	1	0	1	0	0	1	1	0	11	0.52	0	0	0	1	0	1	-0.53	4.80	0
1	1	0	1	0	1	0	1	0	0	0	10	0.18	1	1	0	1	0	3	1.54	14.40	0
0	0	0	0	0	0	0	0	1	1	0	6	-1.21	0	0	0	0	0	0	-1.56	0.00	0
0	0	0	1	0	0	0	0	1	1	0	6	-1.21	0	0	0	0	0	0	-1.56	0.00	0
0	0	0	1	0	1	0	0	0	1	0	9	-0.17	0	0	1	1	0	2	0.51	9.60	0
0	0	0	1	0	1	0	0	1	1	0	9	-0.17	0	0	1	1	0	2	0.51	9.60	0
0	0	0	1	0	1	0	0	1	1	0	7	-0.86	0	0	0	0	0	0	-1.56	0.00	0
1	0	0	1	0	1	0	1	0	1	1	12	0.87	0	0	1	1	0	2	0.51	9.60	0
0	0	0	1	0	1	0	0	0	1	0	6	-1.21	0	0	0	0	0	0	-1.56	0.00	0
0	0	0	1	0	1	0	0	1	1	0	7	-0.86	0	0	0	1	0	1	-0.53	4.80	0
1	1	0	1	0	1	0	0	1	0	0	6	-1.21	1	0	1	0	1	3	1.54	14.40	0
1	0	0	1	0	1	0	0	0	1	0	8	-0.52	0	0	0	1	0	1	-0.53	4.80	0
1	0	0	1	1	1	0	0	1	1	1	12	0.87	0	0	0	1	0	1	-0.53	4.80	0
0	0	0	1	1	1	0	1	0	1	0	12	0.87	0	1	0	1	0	2	0.51	9.60	0
0	1	0	1	0	1	0	0	1	0	0	11	0.52	0	0	1	0	0	1	-0.53	4.80	0
1	1	0	1	1	1	0	1	0	0	0	12	0.87	1	0	1	1	0	3	1.54	14.40	1
1	1	0	1	0	0	0	1	1	1	1	8	-0.52	0	0	0	0	0	0	-1.56	0.00	1
0	0	0	1	0	1	0	0	0	0	0	9	-0.17	0	0	0	0	0	0	-1.56	0.00	1
0	1	0	0	0	0	0	0	0	0	0	4	-1.91	0	0	1	1	0	2	0.51	9.60	0
0	0	0	1	1	1	0	0	0	1	0	8	-0.52	1	0	1	1	0	3	1.54	14.40	1
1	0	0	1	0	1	0	1	0	1	1	12	0.87	0	0	0	1	0	1	-0.53	4.80	1
0	0	0	1	0	1	0	0	1	1	0	10	0.18	0	0	1	0	0	1	-0.53	4.80	0
1	0	0	1	0	1	0	0	0	1	1	9	-0.17	0	0	1	0	0	1	-0.53	4.80	0
0	1	0	0	0	0	1	0	1	1	0	8	-0.52	0	0	0	0	0	0	-1.56	0.00	0
0	1	0	1	0	1	0	0	1	1	0	8	-0.52	0	0	0	1	1	2	0.51	9.60	0
0	0	0	1	0	1	0	0	0	1	0	9	-0.17	0	1	0	1	0	2	0.51	9.60	0
0	0	0	1	0	0	1	0	0	1	0	12	0.87	1	0	0	1	0	2	0.51	9.60	0
0	0	0	1	0	1	0	0	0	0	0	7	-0.86	1	0	0	0	0	1	-0.53	4.80	1
0	0	0	1	0	1	1	0	0	0	0	8	-0.52	1	0	1	0	0	2	0.51	9.60	1
0	1	0	1	1	1	0	0	0	0	0	6	-1.21	0	0	0	1	0	1	-0.53	4.80	0
0	0	0	1	1	1	0	0	0	1	0	5	-1.56	0	0	0	1	0	1	-0.53	4.80	0
0	0	0	1	0	1	0	0	0	0	0	6	-1.21	0	0	0	1	0	1	-0.53	4.80	1
0	0	0	1	0	1	1	0	0	1	0	10	0.18	0	0	1	1	1	3	1.54	14.40	0
0	1	0	0	0	0	0	0	0	0	0	5	-1.56	0	0	0	1	0	1	-0.53	4.80	1
0	0	0	1	0	1	1	1	1	0	0	9	-0.17	0	0	1	1	0	2	0.51	9.60	1
0	0	1	1	1	1	0	1	0	1	1	11	0.52	0	0	1	1	1	3	1.54	14.40	0
0	0	0	1	0	1	0	0	0	1	0	9	-0.17	0	0	0	1	0	1	-0.53	4.80	1
0	1	0	1	1	1	1	0	0	1	0	11	0.52	0	0	0	1	0	1	-0.53	4.80	1
0	1	0	1	1	1	0	0	0	1	0	13	1.22	0	0	1	1	0	2	0.51	9.60	1
0	1	0	1	0	1	0	0	0	1	0	11	0.52	0	0	0	0	0	0	-1.56	0.00	0
0	1	0	0	0	0	0	0	0	1	0	8	-0.52	1	0	0	1	0	2	0.51	9.60	0
0	0	0	1	0	1	0	0	0	0	0	8	-0.52	1	0	0	1	0	2	0.51	9.60	0
1	0	0	1	0	1	0	0	0	1	0	13	1.22	0	0	1	0	0	1	-0.53	4.80	1
1	0	0	1	0	1	1	0	0	1	1	15	1.91	1	0	1	1	0	3	1.54	14.40	0
0	1	0	1	0	1	1	0	1	1	0	9	-0.17	0	0	0	1	0	1	-0.53	4.80	1
0	1	0	1	0	1	0	0	0	1	0	7	-0.86	1	0	0	1	1	3	1.54	14.40	0
0	0	0	1	1	1	0	0	1	1	1	9	-0.17	0	0	0	0	0	0	-1.56	0.00	0
0	0	0	1	0	1	0	0	1	1	0	12	0.87	0	0	1	1	0	2	0.51	9.60	0
0	1	0	0	0	0	0	0	0	1	0	5	-1.56	0	0	0	1	0	1	-0.53	4.80	0
0	1	0	1	0	1	0	0	1	1	0	9	-0.17	0	0	1	1	0	2	0.51	9.60	0
1	1	0	1	0	1	1	1	1	1	1	16	2.26	0	0	1	1	0	2	0.51	9.60	1
0	1	0	1	0	1	0	0	1	0	0	9	-0.17	1	0	0	1	0	2	0.51	9.60	0
1	1	0	1	0	1	0	0	1	1	0	9	-0.17	0	0	1	0	0	1	-0.53	4.80	0
1	1	0	1	0	1	1	0	1	1	0	11	0.52	0	0	1	1	0	2	0.51	9.60	1
1	1	0	1	0	1	0	0	1	1	0	13	1.22	1	0	1	1	0	3	1.54	14.40	1
0	0	0	1	0	1	0	0	1	1	0	9	-0.17	0	0	1	0	0	1	-0.53	4.80	0
0	0	0	1	0	1	0	0	1	1	0	7	-0.86	0	0	1	1	0	2	0.51	9.60	0

0	0	0	1	0	1	1	0	0	0	0	8	-0.52	1	0	0	1	0	2	0.51	9.60	0
1	0	0	1	1	0	0	0	0	1	1	6	-1.21	1	0	0	0	1	2	0.51	9.60	0
1	0	0	1	0	1	0	1	0	1	0	10	0.18	0	0	0	1	0	1	-0.53	4.80	0
1	0	0	1	0	1	0	0	1	1	1	14	1.57	0	0	0	0	0	0	-1.56	0.00	0
0	1	1	1	1	0	0	0	1	1	0	9	-0.17	0	0	0	1	1	2	0.51	9.60	1
1	0	0	1	0	1	1	1	1	1	0	15	1.91	0	0	1	1	0	2	0.51	9.60	0
0	0	0	1	0	1	0	0	0	0	0	8	-0.52	0	0	0	0	0	0	-1.56	0.00	1
1	1	0	1	1	1	0	1	1	1	1	14	1.57	0	0	1	1	0	2	0.51	9.60	1
1	1	0	0	0	0	0	1	1	1	0	11	0.52	0	0	0	0	0	0	-1.56	0.00	0
1	0	0	1	0	1	0	0	1	1	0	12	0.87	1	0	1	0	0	2	0.51	9.60	0
1	1	0	1	0	1	1	1	0	0	0	10	0.18	0	0	1	1	0	2	0.51	9.60	1
0	1	0	1	0	0	0	0	0	0	0	8	-0.52	0	0	1	0	0	1	-0.53	4.80	0
0	1	0	1	1	1	0	0	0	1	0	13	1.22	0	0	0	1	0	1	-0.53	4.80	0
0	0	0	1	0	1	0	0	0	1	0	11	0.52	0	0	0	1	0	1	-0.53	4.80	0
1	0	0	1	0	1	0	0	1	1	1	13	1.22	0	0	1	0	1	2	0.51	9.60	1
1	1	0	1	1	0	0	0	1	0	0	10	0.18	0	0	0	1	0	1	-0.53	4.80	0
0	1	0	1	1	1	1	1	1	1	0	14	1.57	0	0	0	0	1	1	-0.53	4.80	1
1	0	0	1	0	1	0	0	0	1	0	9	-0.17	0	0	1	1	0	2	0.51	9.60	0
0	1	0	1	0	1	1	0	0	1	0	12	0.87	0	0	0	0	0	0	-1.56	0.00	1
0	1	0	1	0	1	0	0	0	1	0	7	-0.86	0	0	0	1	0	1	-0.53	4.80	1
0	1	0	1	0	1	0	0	0	1	0	7	-0.86	0	0	0	0	0	0	-1.56	0.00	0
0	0	0	1	0	1	0	0	1	1	0	9	-0.17	0	0	0	1	0	1	-0.53	4.80	0
0	1	0	1	0	1	0	0	1	0	0	9	-0.17	0	0	0	0	0	0	-1.56	0.00	0
0	1	0	1	0	1	0	0	1	1	0	8	-0.52	0	0	0	0	0	0	-1.56	0.00	0
1	1	0	1	1	1	0	1	1	1	1	17	2.61	0	1	1	0	0	2	0.51	9.60	0
1	0	0	1	0	1	0	0	0	1	1	9	-0.17	1	0	1	0	0	2	0.51	9.60	0
1	0	0	1	1	1	0	1	1	1	1	14	1.57	1	0	1	0	0	2	0.51	9.60	0
1	1	0	1	0	1	1	0	1	1	1	15	1.91	0	0	1	0	0	1	-0.53	4.80	0
1	0	0	1	0	1	0	0	1	1	0	15	1.91	0	0	1	1	1	3	1.54	14.40	0
0	1	0	1	0	1	1	0	1	0	0	7	-0.86	0	0	0	1	0	1	-0.53	4.80	0
0	1	0	1	0	1	0	1	0	1	0	10	0.18	0	0	1	1	1	3	1.54	14.40	0
0	0	0	1	0	1	0	0	1	0	0	8	-0.52	1	0	1	0	0	2	0.51	9.60	0
1	0	0	1	0	1	0	0	1	1	0	14	1.57	1	0	1	1	0	3	1.54	14.40	0
0	1	0	1	0	1	0	1	1	1	0	11	0.52	0	0	0	0	0	0	-1.56	0.00	0
0	0	0	1	0	1	1	0	0	1	1	8	-0.52	0	0	1	1	1	3	1.54	14.40	0
1	0	0	1	0	1	1	1	1	1	1	13	1.22	1	0	1	1	1	4	2.57	19.20	1
0	1	0	1	0	1	0	1	1	1	0	14	1.57	1	0	1	0	0	2	0.51	9.60	0
1	0	0	1	0	1	0	1	0	0	0	9	-0.17	0	0	1	1	1	3	1.54	14.40	0
0	0	0	1	0	1	0	0	1	0	0	8	-0.52	0	0	1	0	0	1	-0.53	4.80	0
0	0	0	1	0	1	1	0	0	0	0	11	0.52	0	0	0	1	0	1	-0.53	4.80	1
0	1	0	1	1	1	0	0	1	1	0	12	0.87	0	0	0	1	0	1	-0.53	4.80	0
0	1	0	1	0	1	0	0	1	0	0	9	-0.17	0	0	1	0	0	1	-0.53	4.80	0
1	1	0	1	0	1	0	0	1	0	0	12	0.87	0	0	1	0	0	1	-0.53	4.80	0
1	0	0	1	0	1	0	0	1	1	1	12	0.87	1	0	1	1	0	3	1.54	14.40	1
1	0	0	1	1	1	1	1	1	0	0	13	1.22	1	0	1	1	0	3	1.54	14.40	1
1	1	0	1	1	1	1	1	0	0	0	13	1.22	1	0	0	1	0	2	0.51	9.60	1
1	0	0	1	1	1	0	0	0	1	1	12	0.87	0	0	1	0	0	1	-0.53	4.80	1
0	0	0	1	0	1	0	1	0	1	1	10	0.18	1	0	1	0	1	3	1.54	14.40	0
0	0	0	1	0	1	1	0	1	1	0	11	0.52	0	0	0	0	0	0	-1.56	0.00	0
0	1	0	1	0	1	1	0	1	1	0	10	0.18	0	0	0	1	0	1	-0.53	4.80	0
1	1	0	1	0	1	1	1	0	0	0	13	1.22	0	0	1	1	0	2	0.51	9.60	1
1	0	0	1	0	1	0	0	0	1	1	9	-0.17	1	0	1	0	0	2	0.51	9.60	1
1	1	0	1	0	1	0	1	1	1	0	13	1.22	0	0	0	1	0	1	-0.53	4.80	1
0	1	0	1	0	1	1	1	0	1	0	10	0.18	0	0	0	1	0	1	-0.53	4.80	0
1	1	0	1	0	1	0	0	1	1	0	11	0.52	0	0	0	1	1	2	0.51	9.60	0
1	1	0	0	0	0	0	1	0	0	0	8	-0.52	0	0	1	0	0	1	-0.53	4.80	0
0	1	0	1	0	1	0	0	0	1	0	11	0.52	1	0	1	0	0	2	0.51	9.60	0
1	0	0	1	1	1	0	0	0	1	0	11	0.52	1	0	0	1	1	3	1.54	14.40	0
1	1	0	1	0	1	0	0	0	1	1	12	0.87	1	0	1	1	0	3	1.54	14.40	1
0	0	0	1	0	0	1	1	0	1	0	9	-0.17	1	0	0	1	1	3	1.54	14.40	0
0	0	0	1	0	1	1	0	1	1	0	10	0.18	0	0	0	0	0	0	-1.56	0.00	1
0	1	0	0	0	0	1	0	0	1	0	9	-0.17	0	0	0	1	1	2	0.51	9.60	1
0	1	0	1	0	1	1	0	0	1	0	9	-0.17	1	0	0	1	0	2	0.51	9.60	1
1	1	0	0	0	0	0	1	0	1	1	12	0.87	1	0	1	1	0	3	1.54	14.40	1
0	1	0	1	0	1	0	1	0	1	0	8	-0.52	0	0	0	1	1	2	0.51	9.60	1
1	0	0	1	0	1	0	0	0	1	1	10	0.18	1	0	1	0	0	2	0.51	9.60	1
0	0	0	1	0	1	1	0	0	1	0	8	-0.52	0	0	1	0	0	1	-0.53	4.80	1

0	0	0	1	0	1	0	0	0	1	0	8	-0.52	0	0	1	0	0	1	-0.53	4.80	1
0	1	0	0	0	0	0	0	1	0	0	7	-0.86	0	0	0	0	0	0	-1.56	0.00	0
1	0	0	1	0	1	0	0	1	1	0	9	-0.17	1	0	1	0	0	2	0.51	9.60	0
1	0	0	1	0	1	1	0	0	1	1	9	-0.17	0	0	0	0	0	0	-1.56	0.00	1
1	1	0	1	0	0	0	0	0	1	1	10	0.18	0	0	1	1	0	2	0.51	9.60	1
0	0	0	1	0	0	0	0	1	1	0	10	0.18	0	0	1	0	1	2	0.51	9.60	0
0	1	0	1	0	1	0	0	1	0	0	7	-0.86	0	0	0	1	0	1	-0.53	4.80	0
1	0	0	1	0	1	0	1	1	0	0	10	0.18	0	0	0	1	0	1	-0.53	4.80	0
1	0	0	1	0	1	0	0	0	1	1	13	1.22	0	0	0	0	0	0	-1.56	0.00	0
1	0	0	1	0	1	0	1	1	1	1	11	0.52	1	1	0	0	1	3	1.54	14.40	0
0	0	0	0	0	0	0	0	0	1	0	4	-1.91	0	0	1	0	0	1	-0.53	4.80	0
1	0	0	0	0	0	0	1	0	1	0	9	-0.17	0	0	1	1	0	2	0.51	9.60	1
0	1	0	1	0	1	0	0	1	1	0	8	-0.52	0	0	0	0	0	0	-1.56	0.00	1
1	0	0	0	1	1	0	0	0	1	0	9	-0.17	0	0	0	0	0	0	-1.56	0.00	1
1	0	0	0	1	1	0	0	0	1	1	14	1.57	1	0	0	0	0	1	-0.53	4.80	0
0	1	0	0	1	0	0	0	0	1	0	9	-0.17	0	0	1	1	0	2	0.51	9.60	0
0	1	0	0	1	0	1	0	0	0	0	7	-0.86	0	0	1	0	0	1	-0.53	4.80	0

VP2	VP3	VP4	VP5	ESCVP	ESCVPp	ESCVp2	RSHVIDA	RSH12M	IST
0	1	0	0	1	-0.83	4.80	1	0	0
0	0	0	0	0	-1.91	0.00	0	0	0
0	1	1	0	2	0.26	9.60	1	1	1
0	0	0	0	0	-1.91	0.00	1	0	1
0	1	0	0	1	-0.83	4.80	0	0	0
0	1	0	0	1	-0.83	4.80	1	0	0
0	1	1	0	2	0.26	9.60	1	0	0
0	1	0	0	1	-0.83	4.80	1	0	0
0	1	1	0	3	1.35	14.40	0	0	0
0	1	1	0	2	0.26	9.60	1	0	0
0	1	1	0	2	0.26	9.60	0	0	1
0	1	1	0	2	0.26	9.60	1	0	0
0	1	1	0	2	0.26	9.60	1	0	1
0	0	0	0	0	-1.91	0.00	1	0	0
0	1	1	0	2	0.26	9.60	1	0	1
0	1	1	0	2	0.26	9.60	1	0	0
0	1	1	0	2	0.26	9.60	1	1	1
0	1	1	0	2	0.26	9.60	1	0	0
0	1	0	0	1	-0.83	4.80	1	0	0
0	1	1	0	2	0.26	9.60	0	0	0
0	1	0	0	1	-0.83	4.80	1	0	0
0	1	0	0	1	-0.83	4.80	1	1	1
0	1	0	0	1	-0.83	4.80	1	0	1
0	0	1	0	1	-0.83	4.80	0	0	0
1	1	1	0	4	2.43	19.20	0	0	0
0	1	0	0	2	0.26	9.60	1	1	1
0	1	1	0	3	1.35	14.40	0	0	0
0	1	1	0	2	0.26	9.60	0	0	0
0	1	0	0	2	0.26	9.60	1	0	0
1	1	1	1	5	3.52	24.00	1	1	1
0	1	1	0	2	0.26	9.60	1	0	1
0	1	1	0	2	0.26	9.60	1	1	0
0	1	0	0	1	-0.83	4.80	1	0	0
0	1	1	0	2	0.26	9.60	1	0	0
0	0	0	0	0	-1.91	0.00	1	0	1
1	1	0	0	2	0.26	9.60	1	0	0
0	1	0	0	2	0.26	9.60	0	0	0
0	0	1	0	2	0.26	9.60	0	0	0
0	1	0	0	1	-0.83	4.80	0	0	0
0	1	1	0	2	0.26	9.60	1	0	0
1	1	1	0	4	2.43	19.20	0	0	0
0	1	1	0	2	0.26	9.60	1	0	0
0	1	1	0	3	1.35	14.40	0	0	0
0	0	0	0	1	-0.83	4.80	0	0	0
0	0	0	0	0	-1.91	0.00	1	1	0
0	1	0	0	2	0.26	9.60	1	0	0
0	0	0	0	1	-0.83	4.80	1	0	1
0	1	1	0	3	1.35	14.40	1	0	1
0	1	0	0	1	-0.83	4.80	1	0	0
0	1	0	0	1	-0.83	4.80	1	0	0
0	1	1	0	2	0.26	9.60	0	0	1
0	1	0	0	2	0.26	9.60	1	0	0
0	0	0	0	0	-1.91	0.00	1	1	1
0	0	0	0	1	-0.83	4.80	1	0	1
0	1	1	0	2	0.26	9.60	1	0	0
0	1	1	0	2	0.26	9.60	1	1	1
0	1	1	0	2	0.26	9.60	1	0	1
0	1	0	0	1	-0.83	4.80	1	0	0
0	1	1	0	2	0.26	9.60	1	0	0
0	1	0	0	2	0.26	9.60	1	1	1
0	1	0	0	1	-0.83	4.80	0	0	1
0	1	0	0	1	-0.83	4.80	1	0	0
0	1	1	0	3	1.35	14.40	1	0	1
0	0	0	0	1	-0.83	4.80	1	0	1
0	1	1	0	2	0.26	9.60	1	0	0
0	1	1	0	2	0.26	9.60	1	0	0

0	1	0	0	1	-0.83	4.80	0	0	1
0	1	0	0	1	-0.83	4.80	1	1	1
0	1	1	0	2	0.26	9.60	1	0	1
0	1	0	0	1	-0.83	4.80	1	1	1
0	1	0	0	2	0.26	9.60	1	0	1
0	1	1	0	2	0.26	9.60	1	0	0
0	1	0	0	2	0.26	9.60	0	0	0
0	1	0	0	2	0.26	9.60	1	1	0
0	1	0	0	1	-0.83	4.80	1	0	0
0	0	0	0	0	-1.91	0.00	1	0	0
1	1	0	0	3	1.35	14.40	0	0	0
0	0	1	0	1	-0.83	4.80	0	0	0
0	1	0	0	1	-0.83	4.80	1	0	1
0	1	0	0	1	-0.83	4.80	1	0	1
0	1	0	0	2	0.26	9.60	1	1	1
0	1	0	0	1	-0.83	4.80	0	0	1
0	1	1	0	3	1.35	14.40	1	0	1
0	1	1	0	2	0.26	9.60	1	0	0
0	1	0	0	2	0.26	9.60	1	0	0
0	1	1	0	3	1.35	14.40	1	0	0
0	1	0	0	1	-0.83	4.80	1	0	1
0	1	1	0	2	0.26	9.60	1	0	0
0	1	0	0	1	-0.83	4.80	0	0	0
0	0	1	0	1	-0.83	4.80	1	0	1
0	1	1	0	2	0.26	9.60	1	1	1
0	1	1	0	2	0.26	9.60	1	1	1
0	1	1	0	2	0.26	9.60	1	1	1
0	1	0	0	1	-0.83	4.80	1	1	1
0	1	1	0	2	0.26	9.60	1	0	0
1	1	1	0	3	1.35	14.40	0	0	0
0	1	1	0	2	0.26	9.60	1	0	0
0	1	0	0	1	-0.83	4.80	0	0	0
0	1	0	0	1	-0.83	4.80	1	0	1
1	0	1	0	2	0.26	9.60	1	0	0
0	1	1	0	2	0.26	9.60	1	1	1
0	1	1	0	3	1.35	14.40	1	1	1
0	0	0	0	0	-1.91	0.00	1	0	1
0	1	1	0	2	0.26	9.60	0	0	1
0	0	0	0	0	-1.91	0.00	0	0	1
0	1	0	0	2	0.26	9.60	0	0	1
0	1	0	0	1	-0.83	4.80	1	0	0
1	1	1	0	3	1.35	14.40	0	0	0
1	1	0	0	2	0.26	9.60	0	0	0
0	1	0	0	2	0.26	9.60	1	1	1
0	1	1	1	4	2.43	19.20	0	0	0
0	1	0	0	2	0.26	9.60	0	0	1
0	1	0	0	2	0.26	9.60	1	1	1
0	1	1	0	2	0.26	9.60	1	1	1
0	1	1	0	2	0.26	9.60	1	0	1
0	1	1	0	2	0.26	9.60	1	0	1
0	1	1	0	3	1.35	14.40	0	0	1
0	1	0	0	2	0.26	9.60	1	1	1
0	1	0	0	2	0.26	9.60	1	0	1
0	1	0	0	1	-0.83	4.80	1	0	0
0	1	1	0	2	0.26	9.60	1	0	1
0	1	1	0	2	0.26	9.60	0	0	1
0	1	1	0	2	0.26	9.60	1	0	1
0	0	0	0	0	-1.91	0.00	1	0	1
0	1	1	0	3	1.35	14.40	1	1	1
1	1	0	0	2	0.26	9.60	1	0	1
0	1	1	0	3	1.35	14.40	1	0	1
0	1	1	0	3	1.35	14.40	1	0	0
0	0	1	1	3	1.35	14.40	1	0	0
0	1	0	0	2	0.26	9.60	1	1	1
1	0	1	0	3	1.35	14.40	1	0	1
0	1	1	0	3	1.35	14.40	1	1	1
0	0	0	0	1	-0.83	4.80	1	0	0

0	1	0	0	2	0.26	9.60	1	0	1
1	1	0	0	2	0.26	9.60	0	0	1
0	1	1	0	2	0.26	9.60	1	0	0
0	1	0	0	2	0.26	9.60	1	1	0
0	1	1	0	3	1.35	14.40	1	1	0
0	0	0	0	0	-1.91	0.00	1	0	1
0	0	1	1	2	0.26	9.60	0	0	0
0	1	0	0	1	-0.83	4.80	0	0	0
0	1	1	0	2	0.26	9.60	1	1	1
0	0	1	0	1	-0.83	4.80	1	1	0
0	1	1	1	3	1.35	14.40	1	0	0
0	1	1	0	3	1.35	14.40	1	0	0
0	1	1	0	3	1.35	14.40	1	0	0
0	1	0	0	2	0.26	9.60	1	0	0
0	1	0	0	1	-0.83	4.80	1	1	1
0	1	1	1	3	1.35	14.40	1	0	1
1	1	0	0	2	0.26	9.60	0	0	0

ID	idade
IND_BRANCA	cor_branca
ESC	escolaridade
UES	tem_uniao_estavel
AR	atividaderemunerada
RPC	renda_per_capta
REL	religiao
VI1	≤ 24 anos
VI2	não_branca
VI3	escol_menor8 anos
VI4	nao_uniao_estavel
VI5	freq_SSapenasAOadoecesentealgumdesconforto
VI6	naotemconhecimentobásicos_ IST/aids
VI7	teveIST
VI8	Nuncarealizousorologias
VI9	naoperceberisco_ IST
VI10	naoperceberisco_ HIV
VI11	PraticasexoAposdroga/alcool
VI12	coitarca ≤ 14anos
VI13	parceriaeventual_ultimo ano
VI14	≥ 2parceiros12meses
VI15	UltimasParcerias_RShomem
VI16	sexoemtroca
VI17	penetracaovaginal_anal
VI18	penetracaanal
VI19	naousapreservativo
VI20	Pratica_tribadismo
VI21	relacaomenstruada
VI22	abusosexual
VI23	alteracao_microbiotavag
VI24	relacao_homem_vida
VI25	relacao_homem_12meses
VS1	naotematividaderemunerada
VS2	temsalariomenor291
VS3	naoeclaraterparceriamulher_SS
VS4	preconceito
VP1	NaoFrequenta_AB
VP2	Não_Disp_CO_MSM
VP3	Não_informacao_IST/aids_SS
VP4	dificuldade_estrutura_SS
VP5	teve_dificuldadeSS_MSMprof
RSHVIDA	relacao_homem_vida
RSH12M	relacao_homem_12meses
IST	TerIST
