# Supplementary material for: Fuzzy Logic: vulnerability of women who have sex with women to sexually transmitted infections
Source: Rev Bras Enferm. 2024 Jul 29;77(3):e20230271. doi: 10.1590/0034-7167-2023-0271 (PMC11290739; doi:10.1590/0034-7167-2023-0271)
Supplement: Translation

# Translation: Lógica *Fuzzy*: vulnerabilidade de mulheres que fazem sexo com mulheres às infecções sexualmente transmissíveis

**DOI:** 10.1590/0034-7167-2023-0271pt

**Authors:** Juliane Andrade, Kendra Yanne da Silva Santos, Ana Paula Freneda de Freitas, Mariana Alice Oliveira Ignácio, Emmanuel Zullo Godinho, Marli Teresinha Cassamassimo Duarte

**Affiliations:** I Universidade Estadual Paulista “Júlio de Mesquita Filho”, Faculdade de Medicina de Botucatu. Botucatu, São Paulo, Brasil; II Hospital Ortopédico e de Medicina Especializada. Brasília, Distrito Federal, Brasil; III Universidade Estadual Paulista “Júlio de Mesquita Filho”, Unidade Auxiliar da Faculdade de Medicina de Botucatu, Centro de Saúde Escola. Botucatu, São Paulo, Brasil; IV Centro Universitário Sagrado Coração, Departamento de Agronomia. Bauru, São Paulo, Brasil

**Keywords:** Lógica *Fuzzy*, Infecções Sexualmente Transmissíveis, Mulheres Que Fazem Sexo Com Mulheres, Vulnerabilidade em Saúde, Saúde Sexual e Reprodutiva

## Abstract

**Objetivo:**

descrever a possibilidade de aplicação da Lógica Fuzzy na análise da vulnerabilidade de Mulheres que fazem Sexo com Mulheres às Infecções Sexualmente Transmissíveis/HIV/aids.

**Métodos:**

Desenvolveu-se um sistema de Lógica *Fuzzy* com 17 variáveis de entrada e uma de saída, utilizando dados relacionados à vulnerabilidade em um município localizado no Centro-Oeste do Estado de São Paulo, Brasil.

**Resultados:**

O fator de maior impacto positivo foi a confirmação de que a baixa compreensão sobre Infecções Sexualmente Transmissíveis/HIV/aids está associada a uma maior vulnerabilidade. Por outro lado, a declaração “Não se expor para a profissional de saúde”, onde a pessoa não admite ter relações sexuais com mulheres, teve o menor impacto.

**Conclusões:**

A Lógica *Fuzzy* oportuniza a identificação da vulnerabilidade, expressa pela análise de interação entre as variáveis de cada dimensão. Isso a torna um método promissor para auxiliar na análise da vulnerabilidade de populações específicas.

## INTRODUÇÃO

A Saúde Sexual e Reprodutiva (SSR) de mulheres com práticas homossexuais ainda é pouco estudada. Ressalta-se que as práticas sexuais, homo, hetero e bissexuais não determinam a identidade sexual dos indivíduos (lésbicas, gays e bissexuais). Logo, a fim de considerar as práticas sexuais para que as necessidades em saúde de mulheres com práticas homo/bissexuais tenham visibilidade e recebam cuidado integral, emerge o termo mulheres que fazem sexo com mulheres (MSM)^(1)^.

No Brasil, por décadas, as mulheres lésbicas, por meio do movimento de Lésbicas, Gays, Bissexuais, Travestis e Transexuais (LGBT) e militância de lésbicas feministas e apoiadores, lutam por visibilidade social e política. Esta luta obteve resultados importantes como a Política Nacional de Atenção Integral à Saúde da Mulher (PNAISM)^(2)^, Cadernos de Atenção Básica sobre Saúde Sexual e Saúde Reprodutiva^(3)^, a Política Nacional de Saúde Integral de LGBT^(4)^, dentre outros. Contudo, ainda muitas são as barreiras/desafios para a implantação efetiva das ações de cuidado na rotina dos serviços de saúde.

No campo da SSR o enfoque à prevenção às Infecções Sexualmente Transmissíveis/Vírus da Imunodeficiência Humana/Síndrome da Imunodeficiência Humana (IST/HIV/aids) é primordial para estabelecer as ações de cuidado, além da adoção de nomenclatura abrangente, que auxilie o enfrentamento de estigmas e situações de discriminação. Nessa perspectiva, são inúmeras as vulnerabilidades de MSM, tais como a falta de informação recebida para prevenção das IST/HIV/aids voltadas às práticas homossexuais^(1,5)^, abordagem heteronormativa por profissionais de saúde, frequentemente acompanhada de discriminação e despreparo para o atendimento, diferença de gênero, quebra de sigilo pelos profissionais das informações referentes às pacientes^(6)^, além da inadequação das medidas de prevenção às IST/HIV/aids recomendadas às práticas homossexuais femininas^(7)^.

Outro fator que contribui para a vulnerabilidade de MSM às IST/HIV/aids é a crença que elas são isentas a essas infecções^(5,7)^ tanto por parte delas quanto pelos profissionais da saúde, evidenciando ainda mais o despreparo profissional nesta temática. Tal crença é desmistificada ao entrar em contato com as prevalências de IST/HIV/aids em MSM. Revisão sistemática, recente, com mulheres autodeclaradas lésbicas ou que relataram práticas sexuais com outras mulheres, sobre o risco de IST/HIV/aids e vaginose bacteriana entre o público do estudo, indicou prevalência de IST/HIV/aids curáveis ou tratáveis em mulheres residentes em países de média-baixa renda foi bastante variável, sendo de 2,4% para clamídia, 0,6% para gonorreia, 3,5% para tricomoníase e 0,5% para sífilis. Os autores citam estudos realizados na China, Reino Unido e Irlanda, além de outra revisão sistemática realizada por pesquisadores indianos, os quais indicaram a prevalência de HPV variando entre 11% e 15,6%^(8)^.

Nesse sentido, a vulnerabilidade expressa-se por um conjunto de aspectos individuais e coletivos à maior suscetibilidade de se expor a adoecimentos ou agravos à saúde, e está diretamente relacionada à maior ou menor disponibilidade de recursos para proteção da saúde. Desse modo, a vulnerabilidade está classificada em três dimensões, geralmente articuladas entre si: individual, social e programática^(9)^.

A dimensão individual engloba recursos pessoais, como acesso à informação às IST/HIV/aids e rede de apoio pessoal, e subjetividade intersubjetiva, como valores, crenças e desejos, que podem ou não estar em conflito; já a dimensão social envolve as relações de gênero, entre gerações, econômicas, raciais e étnicas, processos de estigmatização, pobreza, exclusão social; e a dimensão programática, a qual é analisada a partir da perspectiva de quanto e como as instituições governamentais protegem e promovem o direito à saúde^(9)^.

Para analisar a vulnerabilidade de MSM, a utilização da Lógica *Fuzzy* pode ser uma alternativa, uma vez que esta tem sido aplicada na área da saúde e possui a possibilidade de informações linguísticas das variáveis em estudo, considerando a opinião do especialista sobre o fenômeno observado, bem como, pode tratar de grandes quantidades de dados de modo acessível, facilitando a tomada de decisão^(10)^.

Diversas são as teorias que envolvem a Lógica *Fuzzy*, sendo a principal o manuseio das incertezas e das ambiguidades como determinísticas numéricas^(11)^. Sabendo que o grau de incerteza é conhecido como valor de pertinência, por isso, a Lógica *Fuzzy* trabalha com dados e resultados mais próximos da linguagem não matemática e sim da linguagem humanística, como exemplo, “pode chover” ou “aquele homem é alto”^(12)^.

Frente à escassez de estudos que envolvam a temática apresentada e a urgência de pesquisas que forneçam subsídios para a efetividade das políticas de saúde na qualidade do cuidado a esta população, bem como o uso de sistemas baseados em Lógica *Fuzzy* para apoio a tomada de decisão, propôs-se o seguinte estudo. Este vem a somar com o artigo intitulado “Vulnerabilidade de mulheres que fazem sexo com mulheres às infecções sexualmente transmissíveis”^(13)^, objetivando desenvolver um modelo baseado em regras difusas que avaliem os efeitos causados pela variação de vulnerabilidade social, individual e programática na análise da vulnerabilidade de MSM às IST/HIV/aids.

## OBJETIVO

Descrever a possibilidade de aplicação da Lógica *Fuzzy* na análise da vulnerabilidade de Mulheres que fazem Sexo com Mulheres às Infecções Sexualmente Transmissíveis/HIV/aids.

## MÉTODOS

### Aspectos éticos

O estudo mãe que subsidiou este estudo foi submetido e aprovado pelo Comitê de Ética em Pesquisa da FMB-UNESP e recebeu parecer favorável (nº. 820.717) em 20/10/2014. Todas as participantes foram devidamente esclarecidas sobre os objetivos e forma de participação e, para aquelas que aceitaram, foi solicitado assinar o Termo de Consentimento Livre e Esclarecido.

### Tipo de estudo

Trata-se de um estudo que ilustra o desenvolvimento metodológico referente à análise de dados empregando-se a Lógica *Fuzzy* a um conjunto de dados de pesquisa mais ampla^(14)^, que visou avaliar o acesso aos serviços de saúde e à SSR de MSM, descrito com base na ferramenta *Strengthening the Reporting of Observational Studies in Epidemiology* (STROBE).

### Cenário do estudo

Realizado no município de Botucatu, localizado no Centro-Oeste do Estado de São Paulo.

### Procedimentos Metodológicos

Para simular o pensamento humano, as variáveis que indicam maior vulnerabilidade às IST/HIV/aids, na prática da assistência e segundo o conhecimento científico, foram designadas por três especialistas da temática e de acordo com a literatura científica^(15-17)^. Os especialistas foram recrutados por serem profissionais da área da saúde com vasta experiência na assistência e pesquisa sobre IST/HIV/aids entre MSM.

As variáveis de entrada do sistema de classificação *Fuzzy* proposto foram divididas em três grupos, tendo cada grupo uma determinada quantidade de variáveis de entrada, conforme o referencial de vulnerabilidade (individual, social e programática)^(9)^ e elencadas levando em consideração a disponibilidade dos dados.

No grupo da vulnerabilidade individual, foram incluídas as variáveis: idade ≤ 24 anos; duas ou mais parcerias sexuais nos últimos 12 meses; não ter conhecimentos básicos sobre IST/HIV/aids; prática sexual em troca de dinheiro e/ou drogas; não ter sorologias para IST/HIV/aids; relação sexual com homem na vida; não ter percepção de risco para IST/HIV/aids; relação sexual com homem nos últimos 12 meses; tribadismo (práticas sexuais entre mulheres) e sexo após consumo de droga ilícita e/ou bebida alcoólica e relação sexual no período menstrual^(13)^.

As variáveis do grupo da vulnerabilidade social incluem: renda per capita < R$291,00 e não declarar fazer sexo com mulher no serviço de saúde^(13)^.

Por fim, as variáveis da vulnerabilidade programática são: não disponibilização da citologia oncótica; não ter recebido informações sobre IST/HIV/aids nos serviços de saúde; dificuldade de acesso aos serviços de saúde e dificuldade na relação com o profissional de saúde^(13)^.

Na Figura 1 é apresentada a estrutura do sistema *Fuzzy* proposto, incluindo a interface de entrada, o bloco de regras e a interface de saída. A linha de conexão simboliza o fluxo de dados. Assim sendo, todas as possibilidades das variáveis de entrada ativam regras no bloco de regras que, por sua vez, produzem as possibilidades descritas na variável de saída final.

**Figura 1:** Estrutura do sistema *Fuzzy* proposto

O sistema foi desenvolvido a partir de uma correlação das respostas “não” e “sim” de cada dado de pertinência (individual, social e programática), sendo que cada grupo possuía uma determinada quantidade de variáveis, conforme descrito anteriormente, a individual 11, a social 2 e a programática 4.

Assim, as respostas que estão abaixo ou igual a 50% teria como dado de pertinência “não” e acima de 50% “sim”. Os gráficos foram desenvolvidos no formato gaussiana por apresentarem ondulações, facilitando a visualização das mudanças das variáveis dentro da estrutura, isto é, do ponto máximo para o ponto mínimo, além da importância crítica da estrutura, por estudo utilizando este método. A Figura 2 apresenta cinco exemplos de dados de entrada dos 17 gráficos que foram construídos.

**Figura 2:** Exemplos de cinco das 17 variáveis de entrada do modelo desenvolvido

A variável de saída foi denominada “Vulnerabilidade” e é apresentada no sistema proposto com os seguintes dados de pertinência: Baixo [0,0-0,5], Médio [0,25-0,75] e Alto [0,5-1,0], conforme Figura 3. Trata-se de um resultado semelhante ao que o profissional de enfermagem conseguiria obter com sua experiência em campo, mas que aqui conta com o apoio da Lógica.

**Figura 3:** Função de pertinência da variável de saída “Vulnerabilidade”

Neste sentido, a Lógica *Fuzzy* aliada ao referencial teórico de vulnerabilidade de Ayres et al.^9^ permite uma melhor resposta sobre a vulnerabilidade de MSM às IST/HIV/aids, buscando otimizar diversas variáveis aplicadas no dia a dia do paciente. Portanto, a apresentação da aplicação da Lógica *Fuzzy* foi proposta no formato de Estudo de Caso, que incluiu uma participante do estudo mãe.

### Coleta e organização dos dados

Os dados do estudo principal foram coletados por meio da aplicação de um questionário elaborado especificamente para a pesquisa, realização de exame ginecológico e coleta de sangue periférico, conforme detalhado em Andrade et al.^(13)^. A população-alvo do estudo foi constituída por mulheres que declararam fazer sexo com mulheres ou com mulheres e homens, acima de 18 anos, residentes em municípios pertencentes ao Departamento Regional de Saúde de Bauru (DRS-VI). Para o recrutamento das participantes, realizou-se ampla divulgação. Os exames oferecidos visavam detectar o diagnóstico de alguma IST: *Chlamydia trachomatis, Neisseria gonorrhoeae, Trichomonas vaginalis*, infecções por Papiloma Vírus Humano (HPV), Vírus da Imunodeficiência Humana (HIV), hepatite B ou sífilis.

### Análise dos dados

Foi utilizado o método de inferência de Mamdani^(12)^ para calcular os valores numéricos das variáveis de entrada em relação à variável de saída pelo software Matlab 2022b, para que pudesse construir um sistema baseado em regras *Fuzzy* computacionais e desenvolver plotagens e mapas de contorno da função de representação do sistema associado. Salienta-se que o software utilizado tem licença pelo grupo de pesquisa AGROENERBIO - Energia e simulação na engenharia de biossistemas e no agronegócio que fica na USP/FZEA em Pirassununga SP.

O método de Mamdani traz como resposta um conjunto *Fuzzy* originado da combinação dos valores de entrada com os seus respectivos graus de pertinência, por meio do operador mínimo e, em seguida, pela superposição das regras pelo operador máximo^(12)^. Para o processo de defuzzificação, foi utilizado o centro de Área (C-o-A), o qual retorna o centro da área debaixo a curva desenvolvida, utilizado por autores^(11)^.

## RESULTADOS

O quantitativo de regras total foi relacionado com o número de combinações possíveis para as respostas das 17 variáveis de cada mulher, considerando que cada variável tem a opção de duas alternativas, “não” ou “sim”. Assim, as bases de regras relacionam as variáveis de entrada e a variável de saída com proposições do tipo “SE” e “ENTÃO”. Deste modo, somaram-se 103.250 correlações, cujas combinações forneceram a resposta do sistema (Tabela 1).

**Tabela 1:** Base de regras do sistema *Fuzzy*

	1	2	3	4	...	8	9	10	11	12	13	14	15	16	17	1
**Id**	**VE**	**VE**	**VE**	**VE**	...	**VE**	**VE**	**VE**	**VE**	**VE**	**VE**	**VE**	**VE**	**VE**	**VE**	**VS**
1	Sim	Sim	Sim	Sim	Sim	Sim	Sim	Sim	Sim	Sim	Sim	Sim	Sim	Sim	Sim	B
2	Sim	Sim	Sim	Sim	Sim	Sim	Sim	Sim	Sim	Sim	Sim	Sim	Sim	Sim	Não	M
3	Sim	Sim	Sim	Sim	Sim	Sim	Sim	Sim	Sim	Sim	Sim	Sim	Sim	Não	Sim	A
4	Sim	Sim	Sim	Sim	Sim	Sim	Sim	Sim	Sim	Sim	Sim	Sim	Sim	Não	Não	B
5	Sim	Sim	Sim	Sim	Sim	Sim	Sim	Sim	Sim	Sim	Sim	Sim	Não	Sim	Sim	B
6	Sim	Sim	Sim	Sim	Sim	Sim	Sim	Sim	Sim	Sim	Sim	Sim	Não	Sim	Não	M
7	Sim	Sim	Sim	Sim	Sim	Sim	Sim	Sim	Sim	Sim	Sim	Sim	Não	Não	Sim	M
8	Sim	Sim	Sim	Sim	Sim	Sim	Sim	Sim	Sim	Sim	Sim	Sim	Não	Não	Não	B
...	...	...	...	...	...	...	...	...	...	...	...	...	...	...	...	...
103.249	Não	Não	Não	Não	Não	Não	Não	Não	Não	Não	Não	Não	Não	Não	Não	M
103.250	Não	Não	Não	Não	Não	Não	Não	Não	Não	Não	Não	Não	Não	Não	Sim	M

*Notas: id: Identificação; VE- Variável de entrada; VS - Variável de saída.*

A estratégia adotada foi a formulação de regras combinando todas as possibilidades das variáveis de entrada em relação à variável de saída. Como exemplo, na primeira linha da Tabela 1, as variáveis de entrada foram todas correlacionadas entre si e tinham respostas “sim” para resultar em uma variável de saída “Baixo”, e assim sucessivamente.

A partir do modelo desenvolvido, foi possível preparar as respostas das superfícies em formato 3D para a vulnerabilidade e seus mapas de contorno, verificando a real inferência dos 17 blocos das variáveis de entrada. Este modelo baseado em regras *Fuzzy* verificou todas as combinações entre as variáveis de entrada, em 2 níveis (“não” e “sim”), resultando em uma base de regras de 103.250 combinações, sendo um fatorial 2^17, conforme modelos de superfície de resposta difusa na Figura 4.

**Figura 4:** Modelos de superfície 3D de Vulnerabilidade em resposta às 17 variáveis de entrada

*Legenda: (Figura a): A: não declara fazer sexo com mulher no serviço de saúde x B: renda per-capita < R$291,00; (Figura b): A: relação sexual com homem nos últimos 12 meses x B: não ter percepção de risco para IST/HIV/aids; (Figura c): A: não ter conhecimentos básicos sobre IST/HIV/aids x B: prática sexual em troca de dinheiro e/ou drogas; (Figura d): A: duas ou mais parcerias sexuais nos últimos 12 meses x B: renda per-capita < R$291,00.*

Compreendeu-se (Figura 4) que o indicador que mais respondeu a uma vulnerabilidade alta foi quando a pessoa possuía baixo conhecimento. Na Figura 4, este indicador representou um valor intermediário entre 0,5 e 1,0, sendo 1,0 o ponto máximo. O que menos impactou a vulnerabilidade da pessoa nesta modelagem foi a declaração de não se expor para a profissional de saúde com 0,1498, mais bem exemplificado, onde a pessoa não declara que faz sexo com mulheres para a profissional de saúde.

De forma mais detalhada, a Figura (4a) apresentou duas regiões importantes, tendo I o ponto com maior vulnerabilidade, quando se avaliou as variáveis de entrada (não declara fazer sexo com mulher no serviço de saúde x renda per capita < R$291,00), pois em ambos tiveram respostas no ponto mais alto (2,0), resultando assim, que ambas as variáveis foram determinantes para se responder a uma alta vulnerabilidade. Já no indicador II, aconteceu o inverso, pontos mais baixos indicam menores chances de vulnerabilidade. Importante salientar que nesta Figura (a), ambas as variáveis possuem preponderância para alta vulnerabilidade, quando os pontos de caracterização estão próximos de 100%.

A Figura (4b) apresentou a relação entre as variáveis relação sexual com homem nos últimos 12 meses x não ter percepção de risco para IST/HIV/aids, sendo que no ponto I, o indicativo de maior vulnerabilidade foi não ter a percepção de risco para IST/HIV/aids, pois ele apresentou tanto “não” como “sim” em pontos fortes, já para a relação sexual com homens nos últimos 12 meses os valores foram acima de 80% para “sim”. Uma característica marcante nesta figura foi que não ter percepção de risco para IST/HIV/aids foi considerado o fator que mais impacta na vulnerabilidade. Para o ponto II, o mesmo ocorre para a variável não ter a percepção de risco para IST/HIV/aids e menor indicativo na variável relação sexual com homens nos últimos 12 meses.

Para a Figura (4c), a alta vulnerabilidade marcada no ponto I, tendo como alto indicador “sim” para prática sexual em troca de dinheiro e/ou drogas, com menor indicador não ter conhecimentos básicos sobre IST/HIV/aids. Para o menor indicador de vulnerabilidade foi ter um amplo conhecimento sobre as duas variáveis relacionadas. Esta Figura (4c) não teve as características das figuras anteriores (a e b), pois ambos não representaram uma alta relação para vulnerabilidade.

Na Figura (4d), apresentou-se os indicadores de alta e baixa vulnerabilidade (I e II), respectivamente, sendo que no ponto I, o maior indicador foi representado para renda per capita < R$291,00, pois pode levar a uma falta de informação importante para prevenção do adoecimento. Quando se avaliou o ponto II, ambos os indicadores estiveram nos pontos mais altos, resultando em uma baixa vulnerabilidade, isso quando aplicadas as variáveis duas ou mais parcerias sexuais nos últimos 12 meses x renda per capita < R$291,00.

## DISCUSSÃO

Foi possível aplicar a Lógica *Fuzzy*, conforme o passo a passo exemplificado em estudo de caso, para determinar e apoiar em dados científicos uma possível vulnerabilidade de uma MSM às IST/HIV/aids. O uso de ferramentas baseadas em inteligência artificial, como proposto neste artigo, traz contribuições a todos os envolvidos nesta questão, sejam eles profissionais ou usuárias do sistema de saúde. Sendo este um estudo piloto, trata-se de um incentivo para o desenvolvimento de software que facilite a análise de dados voltados ao tema.

A Lógica *Fuzzy* como base para criação de uma tecnologia em saúde permite a tomada de decisão e resolução de problemas complexos no âmbito individual e coletivo do cuidado, agrega a este integralidade, através da articulação com outros setores assistenciais, e efetividade, por meio do planejamento e alcance dos objetivos de acordo com a análise das necessidades em saúde da paciente, da população específica do qual a mesma faz parte ou da população em geral, contribuindo, assim, para minimizar aspectos da vulnerabilidade programática^(18)^.

As análises estatísticas transformam números em informações. Neste artigo, optou-se por utilizar a Lógica *Fuzzy* para tratarmos diretamente as informações próprias da linguagem humana, transformando as respostas das pacientes em situações que retratam sua vulnerabilidade. Apesar deste estudo lidar com respostas binárias, “não” ou “sim”, é possível incorporar diferentes respostas no software da Lógica *Fuzzy* para que a análise seja mais fidedigna, uma vez que, para exprimir a linguagem humana, deve-se compreender as nuances incertas que existem entre o “não” e o “sim”. Trata-se de uma pesquisa que ilustra o uso da Lógica *Fuzzy* para análise de vulnerabilidade. Logo, a importância do presente artigo para a população de MSM, visto ainda os desafios enfrentados, principalmente no que tange sua invisibilidade nos serviços de saúde e despreparo profissional no cuidado a esta população^(13)^.

Deste modo, o sistema de informação proposto pode ser transformado em aplicativo para dispositivos móveis e/ou softwares para computadores, de modo que possam ser utilizados inclusive de forma prognóstica e diagnóstica. As tecnologias em saúde apoiadas na Lógica *Fuzzy* já vêm sendo utilizadas em diversas áreas da saúde, como no acompanhamento de crianças e adolescentes com doenças crônicas através de um Sistema Especialista de Suporte à Decisão^(19)^, e na determinação do grau de risco de saúde da gestante^(20)^.

Recomenda-se, ainda, antes do desenvolvimento do aplicativo, realização de estudo transversal aplicando a Lógica *Fuzzy* ao conjunto de mulheres incluídas no estudo maior^(14)^ a fim de operacionalizar a aplicação do método aqui apresentado.

### Limitações do estudo

Destaca-se como limitação do estudo o fato de as variáveis terem respostas binárias, (“sim” ou “não”), não cabendo dentro da natureza da pesquisa respostas como “talvez”, “muito”, “pouco”, condição esta que poderia ser explorada na Lógica Fuzzy. Ainda podem ser citadas como limitações do estudo a forma de escolha das variáveis de entrada por conveniência, destacando-se que inúmeras outras condições de vulnerabilidade podem coexistir, aumentando a vulnerabilidade das MSM às IST/HIV/aids.

### Contribuições para a enfermagem e saúde publica

A possibilidade de aplicação prática do conceito de vulnerabilidade, por meio do emprego da Lógica *Fuzzy*, com apoio de tecnologias da informação, contribui para a reflexão do cuidado em enfermagem em saúde pública.

## CONCLUSÕES

A Lógica *Fuzzy* pode oportunizar a análise da vulnerabilidade expressa pela interação entre as variáveis de cada dimensão, mostrando-se um método de análise viável para populações específicas. Sugerem-se o desenvolvimento de estudos com amostras populacionais maiores que possam aprimorar o modelo e orientar o desenvolvimento de tecnologias que facilitem a tomada de decisão em cenários reais de prática profissional.

## Data Availability

https://hdl.handle.net/11449/255136
